# Ten pillars for the expansion of health system infection prevention capacity

**DOI:** 10.1017/ash.2024.19

**Published:** 2024-03-14

**Authors:** Lisa K. Sturm, Tina R. Jacobs, Mohamad G. Fakih

**Affiliations:** 1 Quality Department, Ascension, St. Louis, MO, USA; 2 Wayne State University School of Medicine, Detroit, MI, USA

## Abstract

The COVID-19 pandemic has accelerated changes in health care across the nation. Particularly, infection prevention programs have been subjected to pressures and increased responsibilities with no expansion in support. In addition, there is a rapid trend for health systems to merge to ensure long term sustainability. Based on our experience leading infection prevention at one of the largest health systems in the United States, we outline how systems can provide and increase capacity to optimize and enhance the hospital level infection prevention programs and outcomes. In this commentary, “Ten Pillars for the Expansion of Health System Infection Prevention Capacity” we offer 10 categories of what we have found to establish a successful and functioning infection prevention program. The pillars to support the infection prevention programs focus on structure, processes, empowerment, and partnerships, and the elements and strategies that comprise them.

## Introduction

**Figure 1.**
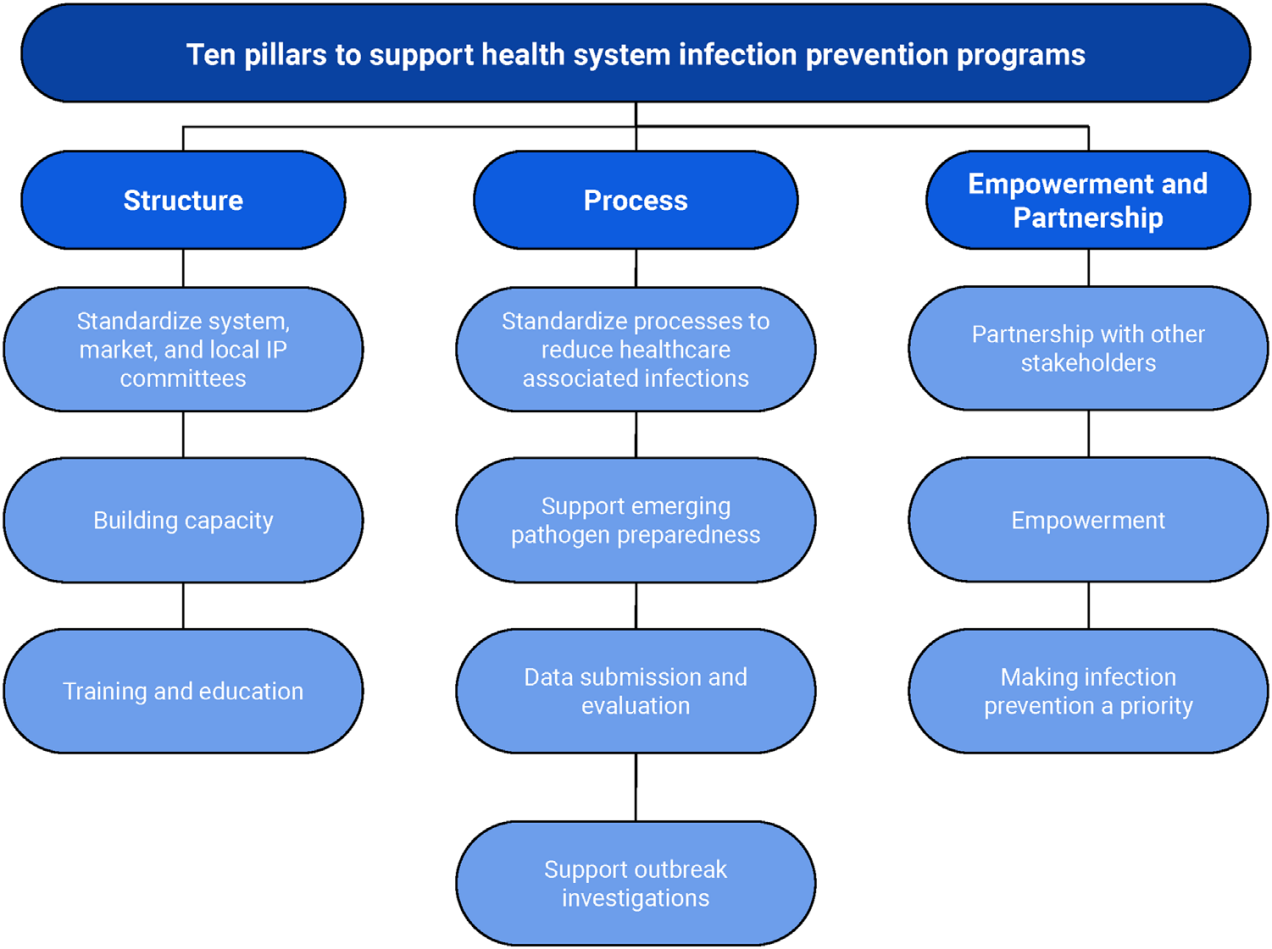
Infection prevention system model based on structure, process, empowerment, and partnership.

The Coronavirus 2019 (COVID-19) pandemic has had a substantial impact on health care in the United States, with a sharp increase in healthcare-associated infections (HAIs),^1–3^ patients with transmissible disease, large disruptions in processes, workflows, supply chain, and an exhausted workforce. Infection preventionists (IP), healthcare epidemiologists (HE), and Infectious Diseases physicians served as experts in preparing and managing the infectious risks posed by the pandemic. They often had to manage the pandemic in addition to their regular daily tasks, in times of uncertainty and evolving recommendations.^4^ Their workload increased, augmented by additional reporting requirements connected to the pandemic, with some reporting up to a 500% increase in calls to IPs.^5^ Many experienced IPs decided to leave the field or retire early^6^ even before the pandemic, resulting in a high turnover^7–9^ and exacerbating shortages. Moreover, there is a paucity of HEs or physicians with formal training to support infection prevention. The pandemic’s heavy emotional impact resulted in extensive IP and HE burnout and turnover with mental health being a large contributing factor.^10,11^ The pandemic has also brought significant changes to the operating healthcare model, with many services migrating from the acute care setting to the ambulatory space, and hospitals becoming home to higher risk and more complex patients. Moreover, hospitals have been experiencing financial struggles further limiting their resources.^12^ Additional economic pressures in health care have also affected the HE and IP workforce and the effectiveness of local infection prevention programs. We outline how a system can provide and increase capacity to optimize and enhance the hospital level infection prevention programs using the example of Ascension, one of the largest non-profit healthcare systems in the United States, spanning across 19 states with nearly 140 hospitals, and more than 2,600 sites of care. We report the ten pillars to support the infection prevention programs focusing on structure, processes, empowerment, and partnerships (Figure 1).

## Program structure

The program was established in 2006 with a physician leader for infection prevention partnering with a system quality director, focusing on few clinical priorities.^13^ Gradually, additional system support was provided recognizing the importance for reducing infection and emerging pathogen preparedness.^14^ A system infection prevention leader position was created (L.K.S.), who reports to the system chief quality officer (M.G.F.) who also functions as the system healthcare epidemiologist and supported by a program manager (T.R.J.). Our system includes 11 geographically distinct regions (markets), each represented by an IP leader and a dyad infectious diseases physician partner. Within the markets, hospital-based IPs typically report to the market IP leader. The role of the system team is to provide support and technical expertise to the market programs to be effective and to reduce the risk of healthcare-associated infections (HAIs).

We address structure in three main areas: standardized infection prevention programs, capacity building, and education/training. Different components support standardizing the function of the infection prevention programs. This includes establishing membership and standing topics for the infection prevention committees for system, market, and hospital levels. For example, the system infection prevention committee has representation from all market IP leaders and their dyad HE (or infectious diseases physicians), nursing, associate health, environmental services, ambulatory, quality, emergency preparedness, analytics, supported by the system HE and IP leader. The system committee sets clear HAI goals that are cascaded to all hospitals. They also develop, review, and approve of core standards or guidelines for patient care, such as central line placement and maintenance, isolation precautions, diagnostic stewardship, and environmental cleaning. We have a system mechanism for product review, evaluation, pilot testing, and selection. Finally, we have one annual risk assessment for respirator fit testing and tuberculosis risk assessments.

To build capacity, the system team also offers a technical resource for the infection prevention programs at the market and hospital levels. Such access to resources helps to build capacity at the hospital level, particularly if the local team members are new or not familiar with what resources are available to them. The system team provides different venues for IPs to stay informed and to be connected to each other as well (Table 1).

**Table 1.** Examples of system supporting structure to engage infection prevention programs

Forum	Structure and purpose
System Infection Prevention and Control Committee	Structured similarly to a hospital committee but with system representatives and all infection preventionist (IP) market leaders and their physician dyads; addresses system priorities for infection prevention, standardization, and plans for improvement
Virtual monthly “all IPs” community calls	Calls available to all IPs (100+ attendees) and include key partners from employee health, regulatory, quality, environmental services. Open chat forum allows for free and easy sharing of ideas, questions and information. Topics: routine updates, celebrations and recognitions, system data updates, and standard work review
Virtual discussion boards centered around the communities	Allow prompt responses to questions and sharing of resources; system IP leader moderates all of them. Multiple forums: IP “all”; IP market leaders; IPs for Ambulatory settings; IPs for Behavioral Health settings; IPs for Pediatric settings
Infection Prevention Internal Internet Resource Page	Access to presentations, resources and toolkits, and other related information

The third area of support under structure is related to training and education. Training and educational needs are assessed from site visits and other sources of feedback and areas of opportunity. System wide programs are then planned based on this information. The offerings are often coordinated at the system level, allowing for ease of access to webinars virtually, free of charge. In addition, we facilitate sharing of resources for training and competencies, from different HE and IP leaders across the system. Those include competency assessments, on-boarding training tools and checklists, and job descriptions.

## Supporting processes

The system supports standardization of infection prevention processes at the market and local levels. The tools and resources include protocols for patient exposures to blood-borne pathogens, position statements on products, and analytics for process and outcome measures for HAIs. There is a system-wide process for collection and dashboard display of National Healthcare Safety Network (NHSN) data at the hospital, market, and system levels.

The COVID-19 pandemic highlighted the value of standardizing processes across the healthcare system. By March 2020, we delivered regular weekly calls attended by all the infection prevention teams, and a standardized toolkit was developed. All routine position statements, guidelines, education, signage, and tools were consistently being generated. Products and environmental standards were put into place, as well as a standard COVID-19 auditing tool, with data submitted to the system team with real-time dashboard displays. Standardized screening tools were also implemented for all sites of care. After the pandemic, we developed and implemented standardized electronic health record entrance screening for travel or infectious diseases of concern for both acute and ambulatory care settings.

The system infection prevention team regularly supports local outbreak investigations, with a rapid response approach via virtual calls. On-site visits can be promptly arranged and evaluation, with written reports and recommendations generated. The system IP leader will in turn share the knowledge and learnings across the markets, with great benefit in improving the care provided.

Data submission, analysis, and display are also standardized system-wide. The system analytics department have a NHSN Group Administrator function for accessing NHSN data. Metrics are agreed upon by the system chief quality officer/HE and IP leader and are displayed in a system dashboard. Data are viewed and downloaded at the system, market, or hospital level. A system “average” is generated for each metric to allow for internal benchmarking. HAI goals are reviewed and established on an annual basis. Discussions with hospitals with opportunities take place, and if necessary, escalation to hospital and/or market leaders takes place. Site visits from the system team may also occur for on-site evaluation and assistance to reduce the HAI.

Leveraging system support has had positive impacts on evaluation of competencies,^15^ device utilization,^16,17^ and enhancement of HAI outcomes.^13,18^

## Supporting empowerment and partnerships

Additional support for infection prevention programs can be manifested through the incorporation of infection prevention goals into organizational priorities, the empowerment of the teams, and building strong partnerships with other stakeholders. For example, we have regularly adopted improving HAIs (eg, central line-associated bloodstream infections, catheter-associated urinary tract infections and hospital-onset methicillin-resistant *Staphylococcus aureus* bacteremia) as system quality priority goals. Additionally, we bundled sepsis improvement efforts with sepsis prevention (preventing infection) and antimicrobial stewardship, keeping the attention to the infection prevention goals. A more recent system-wide initiative during the COVID-19 pandemic to improve quality and safety, “Recognize and Rescue,”^19^ bundled reducing HAIs with efforts to reduce harm events by recognition of high-risk devices, and thus optimized the outcomes of high-risk conditions. Empowerment of the HE and IP is an essential element that the system team fully endorses. Depending on size, many hospitals may not have a HE and may rely on a physician lead with interest in infection prevention. Additionally, on-site IP benefits from having access to a seasoned IP expert. Local IPs are coached and empowered to better achieve their goals and accomplish their initiatives. Moreover, we promote professionalism, encouraging certification and fellowship, presentation in regional and national meetings, and nomination to national awards. Coaching and mentorship are another aspect of support, ensuring the IP team keep the focus on what matters for a success (eg, assistance with annual program risk assessments and prioritization of work). Partnerships with other key stakeholders (Table 2), which allow for even further standardization of work and process, are encouraged, and promoted at the hospital and system levels. This includes close partnership with antimicrobial stewardship teams on diagnostic stewardship and antimicrobial resistance. There is ongoing engagement with environmental services to standardize disinfection products and cleanliness monitoring programs. Other examples include having a standardized Infection Control Construction Risk Assessment tool after development with the Construction Services program.

**Table 2.** Supporting partnership with other stakeholders

Stakeholders	Engagement
Antimicrobial Stewardship	Antibiotic resistance and diagnostic stewardship
Purchasing/Value Analysis Product-Line	Emerging technology reviews, product standards and cost containment
Environmental Services	Standardization on cleaning and disinfection products and performance (eg, adenosine triphosphate monitoring)
Information Technology	Standardized Infection Prevention Clinical Decision Support and emerging pathogen electronic health record screening
Sterile Processing Department team (SPD)	Standardize SPD policies and equipment
Facilities and Safety team	Standardize construction design standards, water management, and environment of care rounds
Associate Health team	Immunization, communicable disease exposure, blood-borne pathogen exposures, respirator and tuberculosis risk assessments
Accreditation and Regulatory	Infection Control Chapter Standards interpretation, assistance with surveys

In closing, health systems can play an essential role supporting local infection prevention programs with tangible and intangible benefits. The sense of community fosters friendships, trust and reliance that ultimately helps build capacity for the HEs and IPs when staffing is lean or if additional expertise is required. The quick access to resources and tools, key stakeholders and partnerships, and actionable dashboards help enhance performance, process and outcomes. Lastly, and potentially more valuable, the local infection prevention programs are part of a bigger structure that provides support, empowerment, and opens channels for accountability.
